# Predicting Mortality in Patients With “Malignant” Middle Cerebral Artery Infarction Using Susceptibility-Weighted Magnetic Resonance Imaging: Preliminary Findings

**DOI:** 10.1097/MD.0000000000002781

**Published:** 2016-03-03

**Authors:** Shu-Ping Chao, Chia-Yuen Chen, Fong Y. Tsai, Wing P. Chan, Chin-I Chen

**Affiliations:** From the Department of Neurology, Shuang Ho Hospital, Taipei Medical University, New Taipei City, Taiwan (S-PC); Department of Radiology, Wan Fang Hospital (C-YC, WPC), Taipei Medical University; Department of Radiology, School of Medicine, College of Medicine, Taipei Medical University, Taipei, Taiwan (C-YC, WPC); Department of Radiological Sciences, University of California, Irvine, CA (FYT); Imaging Research Center (FYT), Taipei Medical University; Department of Neurology, Wan Fang Hospital (C-IC), Taipei Medical University; and Department of Neurology, School of Medicine, College of Medicine, Taipei Medical University (C-IC), Taipei, Taiwan.

## Abstract

To evaluate malignant middle cerebral artery (MCA) infarction (defined as space-occupying edema in more than 50% to 75% of the MCA territory) on magnetic resonance imaging (MRI) with susceptibility-weighted imaging (SWI) sequence and assess the usefulness of SWI findings, diffusion-weighted imaging (DWI) findings, and apparent diffusion coefficient (ADC) as predictors of clinical outcome.

Data from 16 patients with large MCA infarction previously admitted to our institution between December 2009 and October 2012 were retrospectively collected and analyzed. Within 7 days after stroke onset, 1 neurologist and 1 neuroradiologist estimated the area of infarction on DWI/ADC and extent of prominent vessel sign (PVS) on SWI images using the Stroke Program Early MR Score (SPEMRS). The PVS on SWI was defined as a local prominence of hypointense vessels with either increased vessel number or diameter in the target area, when compared with the number or diameter of the contralateral MCA territory vessels.

Six patients died and 10 survived. Although the DWI/ADC-SPEMRS and clinical profiles were similar between the nonsurvivor and survivor groups, SWI-SPEMRS was significantly lower in the nonsurvivor group (*P* < 0.001).

The area of deoxygenation on SWI in patients with malignant MCA infarction can predict mortality. Lower SWI-SPEMRS is a potentially better predictor of poor outcome than lower DWI-SPEMRS. A larger prospective study is needed to clarify the role of SWI as a therapeutic guide in malignant MCA.

## INTRODUCTION

One of the most devastating forms of ischemic stroke, “malignant” middle cerebral artery (MCA) infarction is associated with a fatality rate of up to 80%, if untreated.^1^ Malignant MCA infarction is defined as space-occupying edema in more than 50% to 75% of the MCA territory. Identification of early predictors of infarct growth and malignant course can help to improve treatment decision-making. Currently, patients at particular risk of malignant MCA infarction are identified based on the presence of mismatch between the area of abnormality on diffusion-weighted imaging (DWI) and perfusion-weighted imaging, reduced cerebral blood flow on single-photon-emission CT, and decreased cerebral oxygen metabolism on positron emission tomography and microdialysis.^2–4^ However, most of these procedures are relatively expensive, time-consuming, and not always feasible.

Susceptibility-weighted imaging (SWI) is a noninvasive, fast, high-spatial-resolution, 3-dimensional gradient-echo magnetic resonance imaging (MRI) technique that utilizes postprocessing reconstruction of phase information to accentuate the paramagnetic properties of blood products and thereby contrast.^5^ It is highly sensitive in detecting intravascular venous deoxygenated blood and extravascular blood products.^6^ Cerebral ischemia results in impaired oxygen metabolism due to inadequate perfusion in relation to the high oxygen demands of cerebral tissue. Cerebral infarction is linked to an increase in deoxyhemoglobin concentration due to an imbalance between cerebral oxygen supply and demand in the infarct area. The presence of prominent vessel sign (PVS) on SWI has been reported to reflect an increase in the oxygen extraction fraction compensating for a decrease in arterial cerebral blood flow in acute cerebral infarction, and to be closely related to the amount of deoxyhemoglobin in the veins and capillaries draining the ischemic tissue.^7^

SWI can predict the extent of impaired perfusion in ischemic brain tissue indirectly by focusing on the venous aspect, while DWI and ADC correlate with the usually irreversibly injured ischemic core. A match between SWI and DWI suggests that the brain tissue at risk is already infarcted.^8^

The aim of our study was to determine whether the extent of the PVS evident on SWI could serve as a predictor of clinical outcome in acute malignant MCA infarction.

## PATIENTS AND METHODS

The study protocol was reviewed and approved by the Institutional Review Board of Taipei Medical University. From the stroke registry in our hospital, we retrieved data on consecutive patients with acute stroke admitted between December 2009 and October 2012. Patients were included if they completed the stroke MRI protocol, and had acute large MCA infarction (infarct area more than 50%) identified as hyperintensities on DWI, a complete DWI sequence, and PVS identified on SWI no later than 7 days after symptom onset and confirmed as hypointensities on an apparent diffusion coefficient (ADC) map. Patients were excluded if they had nonlarge MCA infarcts, parenchymal hemorrhage, or hemicraniectomy, or if image quality was poor.

A total of 16 patients (6 men, 10 women; median age 78 years; range, 58–89) met the study criteria and provided their written informed consent. Patients were divided into nonsurvivors (ie, those who died as a result of infarction during admission) and survivors (those who were followed up at least 2 months and 1 patient who was discharged 2 weeks after admission without active medical problems). Risk factors for ischemic stroke, including diabetes mellitus, hypertension, hyperlipidemia, arrhythmia, coronary artery disease, congestive heart failure, smoking, and old cerebrovascular accident were obtained by chart review.^9^

### Imaging Techniques

In the present study, we used a 1.5-T MR system (Magnetom Avanto; Siemens Medical Solutions, Erlangen, Germany) equipped with a standard 12-channel head coil. The following sequences were run: axial T1 spin echo, T2 fast spin echo, fluid attenuated inversion recovery (FLAIR), DWI, and SWI. A slice thickness of 5 mm, gap of 1 mm, and matrix of 256 × 206 pixels were used in all these sequences. Single-shot echo-planar DWI was performed with the following parameters: repetition time/echo time (TR/TE) 3700/109, slice thickness 5 mm, bmax 1000 second/mm^2^, and matrix 128 × 128 pixels. The trace images and ADC maps were computed. The magnitude and phase images of the 3-dimensional SWI sequence were obtained with the following parameters: TR/TE 49/40, slice thickness 2 mm with 64 slices in a single slab, flip angle 15°, iPAT factor 2, and matrix 224 × 256 pixels. The image-processing sequence was adapted from Haacke et al^10^ and performed automatically by a Siemens scanner. The picture archiving and communication system was used to upload data and retrieve the phase, minimal intensity projection, and magnitude of SW images. The total scan time for each subject was less than 30 minutes.

### Data Analysis

The images of selected subjects were retrospectively reviewed independently by 1 senior neurologist (C-IC) and 1 senior neuroradiologist (CYC) who were blinded to the clinical data and to each other's findings. Differences in areas of acute large infarcts measured by the 2 observers on DWI within 7 days after stroke onset were resolved by consensus. The definition of an acute infarct was an area with high signal intensity on DWI and low signal intensity on ADC. The infarct extent was estimated using the Alberta Stroke Program Early CT Score (ASPECTS) modified for MRI (SPEMRS). ASPECTS is a validated 10-point quantitative topographic CT scoring system with the reliability and accuracy needed to assess the extent of the infarct area and forecast outcome in patients with acute large MCA stroke.^11^ In this topographic system, one point is subtracted from the initial score of 10 if there is evidence of infarction in a region. The location of the infarct is recorded as C (caudate nucleus), L (lentiform nucleus), IC (internal capsule), I (insula), M1–3 (lower MCA territory), and M4–6 (higher MCA territory).^8^ This is a negative ordinal scale in which an infarct involving the entire MCA territory is rated 0 and no hyperintensity on DWI/ADC is rated 10. Compared with ASPECTS, SPEMRS is a more quantitative measure of early ischemic change on brain MRI scans.

The PVS on SWI was defined as a local prominence of hypointense vessels with either increased vessel number or diameter in the target area, when compared with the number or diameter of the contralateral MCA territory vessels.^12^ Consensus by both observers was reached.

**FIGURE 1:**
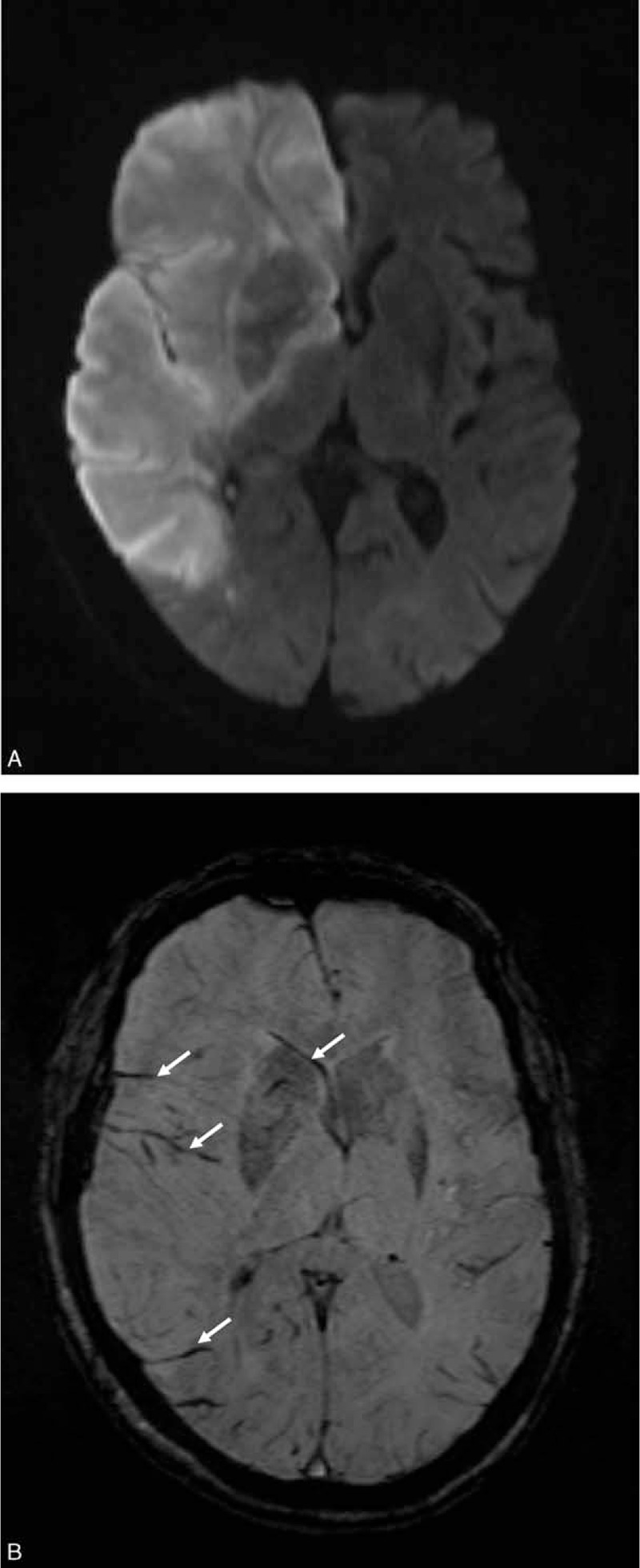
In a 79-year-old woman who died from acute right middle cerebral artery infarction, MRI was performed 2 days after symptom onset. (A) Diffusion-weighted images show hyperintensity in the right cerebral hemisphere including the frontal, parietal, and temporal lobes; insula; basal ganglia; and internal and external capsules. The Stroke Program Early MR Score (SPEMRS) was 0. (B) On susceptibility-weighted imaging, the SPEMRS value was 0 as asymmetric prominent vessels (arrows) were in the insula, internal capsule, caudate nucleus, lentiform nucleus, and all areas of the middle cerebral artery territory.

**FIGURE 2:**
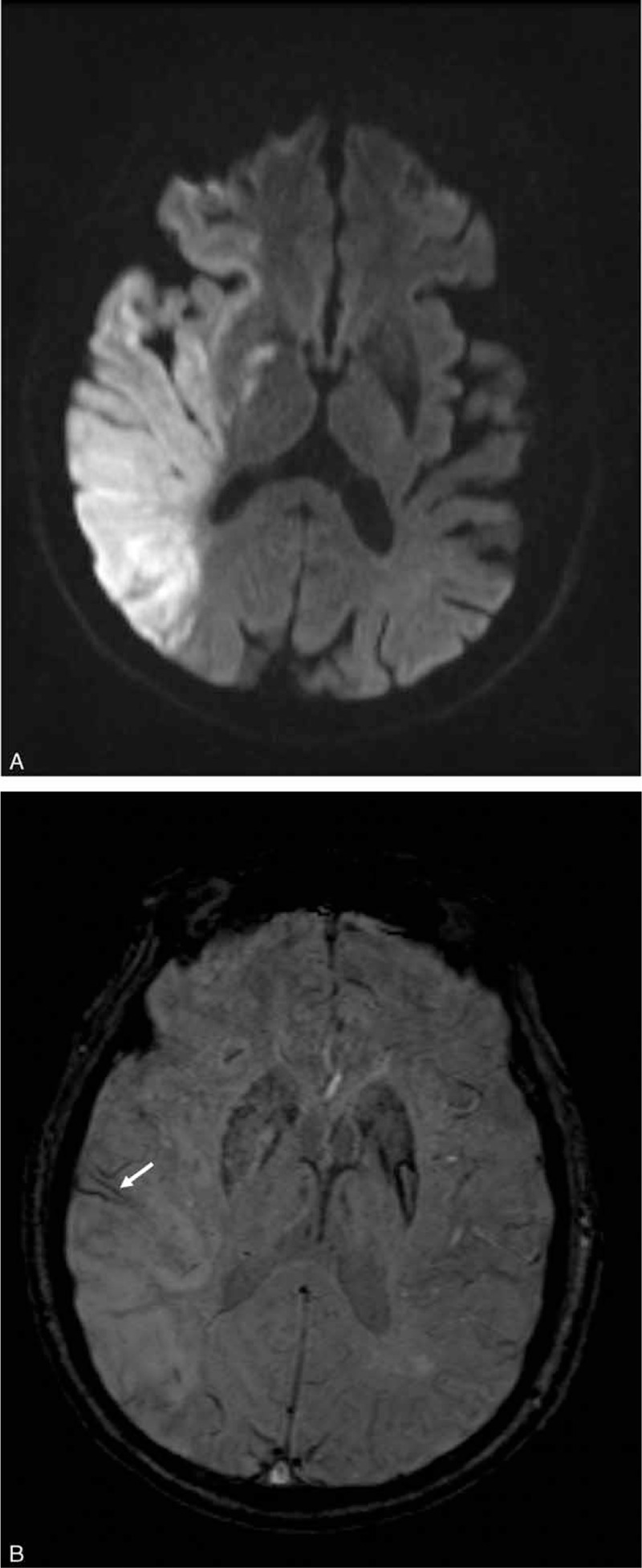
A 76-year-old man who survived after acute right middle cerebral artery infarction. (A) Diffusion-weighted images reveal hyperintensity in the right frontoparietal and temporal lobes; insula; lentiform nuclei; caudate nucleus; internal capsule, and corona radiata. The Stroke Program Early MR Score (SPEMRS) was 0. (B) On susceptibility-weighted imaging, the SPEMRS value was 9, inasmuch as asymmetric prominent vessels (arrow) were in an area of the upper middle cerebral artery territory (M2).

The SPEMRS system was used to estimate the extent of the PVS. The MCA territory was divided into 10 zones, as mentioned previously. Because the thalamostriate vein drains the caudate nucleus, internal capsule, and lentiform nucleus, 3 points were deducted if any PVS was in the thalamostriate vein. Two examples are shown in Figures 1 and 2.

### Statistical Analysis

All statistical analyses used SPSS, version 18.0 (SPSS, Chicago, IL). DWI/ADC data and PVS on SWI are expressed as mean ± standard deviation. The Fisher exact test was used for statistical comparison of all parameters except age and SPEMRS. The Mann–Whitney *U* test was used to compare the results of DWI/ADC and extents of the PVS on SWI between the nonsurvivor and survivor groups. A threshold of *P* < 0.001 was used to determine statistical significance.

## RESULTS

The patients’ clinical profiles and stroke risk factors are presented in Table 1. The mean interval between the onset of symptoms and the MRI examinations was 2.75 days (range 1–7). No significant between-group difference in vascular risk factors (except atrial fibrillation [Af]) or stroke etiology was found. Af was more frequent in the survivor group.

**TABLE 1:**
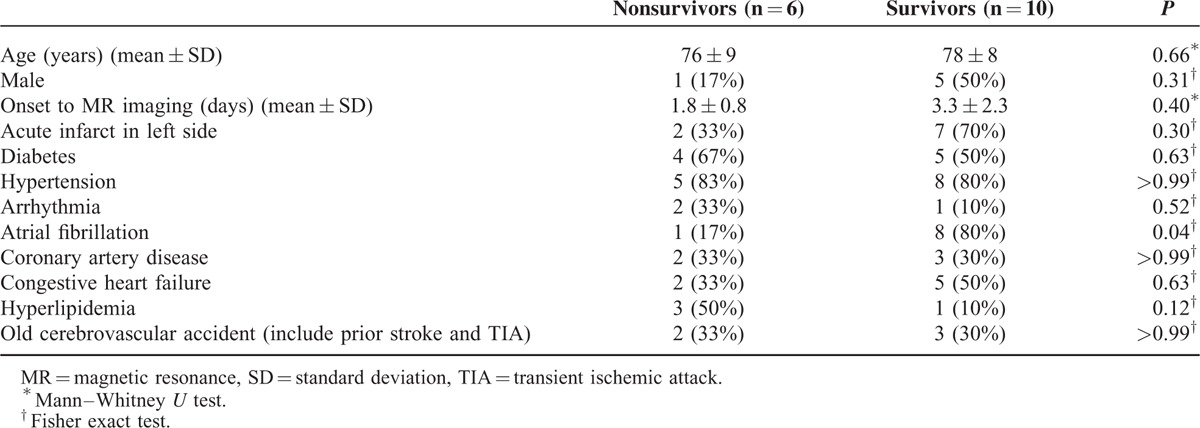
Summary of Clinical Profiles and Stroke Risk Factors

The extent and distribution of the lesions on the DWI/ADC and SWI scans are summarized in Tables 2 and 3. The between-group difference in median SPEMRS value was not significant for DWI/ADC; however, the median SWI-SPEMRS value was significantly smaller in nonsurvivors (2.5 [0–5] vs 10 [7–10]; *P* < 0.001), significantly different between groups for the caudate nucleus, lentiform nucleus, internal capsule, and lower MCA territory (*P* < 0.001), and correlated with outcome.

**TABLE 2:**
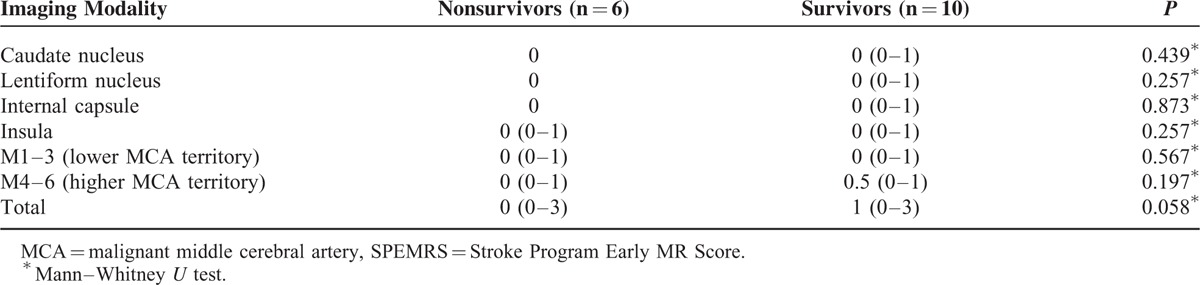
Distribution of Median SPEMRS on Diffusion-Weighted Imaging/Apparent Diffusion Coefficient

**TABLE 3:**
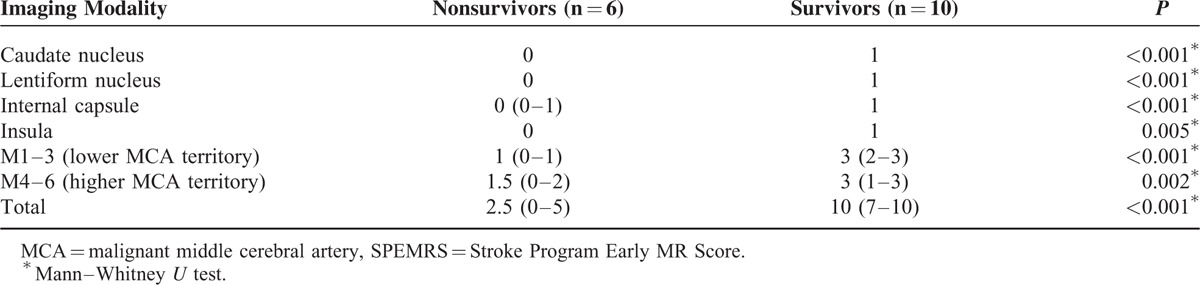
Distribution of Median SPEMRS on Susceptibility-Weighted Imaging

## DISCUSSION

Acute stroke initially causes cellular swelling (cytotoxic edema) which leads to vasogenic edema and infarction. ADC reduction is related directly to the early phase of cytotoxic edema development during ischemia.^13^ Hyperintensity on T2-weighted imaging (T2WI) is indicative of vasogenic edema or infarction. A previous study showed that similar areas of signal intensity on both DWI and T2-weighted images 24 hours after cerebral infarction indicate the coexistence of secondary cytotoxic edema and vasogenic edema. However, compared to T2-weighted imaging findings, the 80% ADC threshold is a better predictor of final infarct volume,^14^ and ADC is a more sensitive measure of the extent of ischemia early after the onset of infarction.^15^ According to Drier et al.^16^ the ADC-based method is as efficient as perfusion-weighted imaging-based methods for estimating infarct growth during the subacute phase.

SWI produces images with very strong T2∗ contrast enhancement and uses both phase and magnitude images to differentiate pial vessels from the background.^17^ Because T2∗-weighted images rarely show the presence of asymmetrically hypointense veins, SWI is a much more suitable candidate sequence^18^ and a better tool to monitor PVS.

In our study, SWI-SPEMRS correlated with outcome in patients with large MCA infarction. Previous studies showed an association of asymmetric deoxygenated cortical vessels and/or transcerebral veins with prognosis in acute stroke patients.^6,12^ Kao et al^19^ found that the perfusion information provided by SWI and by mean transit time methods were similar and that SWI–DWI/ADC mismatch can be a indicator of ischemic penumbra and a predictor of stroke evolution. Mucke et al^20^ identified asymmetric deep medullary veins on SWI as a predictor of stroke severity in patients with acute MCA stroke. Another study also demonstrated that asymmetrical PVS in patients with MCA territory acute ischemic stroke is a strong neuroimaging predictor of 90-day unfavorable prognosis and early neurological deterioration.^21^ Since it signifies oxygen supply and demand imbalance, PVS on SWI is a useful prognosis predictor and SWI-SPEMRS is a useful predictor of outcome in malignant MCA infarction, with lower SWI-SPEMRS indicative of poor outcome.

The main finding of our study is that the extent of PVS on SWI in patients with large MCA infarction can predict mortality. In our study, 6 of 16 patients with malignant MCA infarction had large areas of prominent blood vessels on SWI, all of whom died. A previous report showed that a match between SWI and DWI indicated the brain tissue at risk was already infarcted.^22^ The extent of the deoxygenated area on SWI in malignant MCA infarction can thus predict mortality.

Af was more frequent in our study's survivor group. However, previous studies demonstrated a higher risk of death during the acute phase of stroke in patients with Af than in patients without Af.^23–26^ The reason for this inconsistency between ours and previous studies may be related to small sample size and differences between patient groups. Further study will therefore be needed to clarify this issue.

Limitations of our study included potential bias from small sample size and patient selection. We defined PVS indirectly by comparing the affected side with the contralateral side rather than directly measuring the diameter of the vessel; therefore bias in PVS assessment on SWI existed. The influence of systemic conditions (such as hemoglobin level or oxygen inhalation) is unavoidable. In addition, quantification by the SPEMRS system using SWI is controversial.

To the best of our knowledge, this study is the first to show that SWI-SPEMRS is potentially a better parameter than DWI/ADC-SPEMRS for predicting mortality in patients with malignant MCA infarction. The use of the PVS on SWI as a semiquantitative measure in the early stage of large MCA infarction can predict the patient's outcome with a high degree of diagnostic accuracy.
